# Psychological Factors that Influence Decision-Making Regarding Trauma-Related Pain in Adolescents with Temporomandibular Disorder

**DOI:** 10.1038/s41598-019-55274-9

**Published:** 2019-12-10

**Authors:** Yeon-Hee Lee, Kyung Mi Lee, Tae Kim, Jung-Pyo Hong

**Affiliations:** 1 Department of Orofacial Pain and Oral Medicine, Kyung Hee University Dental Hospital, #26 Kyunghee-daero, Dongdaemun-gu, Seoul, 02447 South Korea; 2 Department of Radiology, Kyung Hee University College of Medicine, Kyung Hee University Hospital, #26 Kyunghee-daero, Dongdaemun-gu, Seoul 02447 South Korea; 3 Department of Biomedical Science and Engineering, Gwangju Institute of Science and Technology, #123 Cheomdangwagi-ro, Buk-gu, Gwangju 61005 South Korea

**Keywords:** Chronic pain, Paediatric research

## Abstract

We evaluated the clinical, magnetic resonance imaging (MRI), and psychological characteristics of adolescents with temporomandibular disorder (TMD) and compared facial macrotrauma effects between young and older adolescents. This case–control study included 70 randomly selected patients (35 young adolescents aged 12–16 years and 35 older adolescents aged 17–19 years) who had been diagnosed with TMD. Each age group was further subdivided according to the presence (T1) or absence (T0) of a macrotrauma history. All patients completed questionnaires on temporomandibular joint (TMJ) pain and dysfunction. We analyzed TMD severity symptoms using TMD-related indexes and the physical changes of TMJ using TMJ MR images. The Symptom Checklist-90-Revised was used to evaluate the patients’ psychological status. Anterior disc displacement was the most frequently observed MRI finding, occurring in a significant proportion of young (47 joints, 67.1%) and older adolescents (40 joints, 57.1%). The prevalence of all the MRI findings (disc displacement, disc deformity, condylar degeneration, and effusion) did not differ between the T0 and T1 subgroups among young and older adolescents. Conversely, the psychological factors differed significantly between the subgroups. Among young adolescents, the mean scores of somatization, obsessive-compulsiveness, hostility, phobic ideation, and psychosis were significantly higher in the T1 subgroup than in the T0 subgroup (all p < 0.05). Furthermore, these increased psychological scores positively correlated with TMD indexes. Clinicians should consider that a weakened psychological status could be an aggravating factor in young adolescents with TMD and should consider the implications in future assessment of such patients.

## Introduction

Temporomandibular disorder (TMD) is an umbrella term that covers heterogeneous clinical problems involving the temporomandibular joint (TMJ), masticatory muscles, or both. The most frequent symptoms are pain in the masticatory muscles and/or in the TMJ, joint sounds, and limitation or deviation of jaw motion^1,2^. TMD is the most common orofacial pain condition of non-dental origin, even though its actual prevalence is a matter of debate^3^. The reported prevalence of TMD ranges from 1% to 75% for objective signs and from 5% to 33% for subjective symptoms^4,5^. Female predominance of TMD has been reported; however, a male predominance of TMD was also reported among patients with a traumatic etiology^6,7^. TMD has numerous causes, including microtrauma due to parafunctional habits and malocclusion, macrotrauma, and stressful conditions^2^. The causes of adolescent TMD have been poorly studied; macrotrauma and psychological impairment are two representative causes^8^, and both should be considered in adolescent TMD, since successful management of TMD is dependent on identifying and controlling the key etiological factors^2^.

The first onset of TMD and development of chronic TMD are fundamentally related to the patient’s age as well as sex. Thus, identification of age-related patterns of TMD symptoms is important. Adolescence is defined as the distinct period covering the transition from childhood to adulthood, and historically, this typically spans from 12 to 19 years of age^9,10^. In accordance with the Research Diagnostic Criteria for Temporomandibular Disorders (RDC/TMD) guidelines and the DC/TMD guidelines, which is the evidence-based new version of the RDC/TMD, patients with TMD under 18 years of age were excluded in many previous studies, owing to the lack of reliability, which has been verified in adult populations^6,11^. Thus, numerous previous TMD studies have focused on adults. A large case−cohort project, named Orofacial Pain: Prospective Evaluation and Risk Assessment (OPPERA), was undertaken to identify the genetic, physiological, psychosocial, and clinical characteristics that influence the development of TMD^12,13^, but was conducted on adults aged over 18 years^14^. However, adolescence is a unique period in which physical, psychological, and socioemotional development occurs simultaneously, and these factors interact with each other in complex ways^9,15^.

Although macrotrauma plays an important role in the development, maintenance, and worsening of TMD symptoms, it has rarely been investigated in adolescents with TMD. Traumatic injuries due to motor vehicle accidents, forceful intubation, third molar extraction, physical abuse, and sports are reported to be etiological factors in the development of TMD^2^. Some recent studies have been performed on adolescent TMD. Among children and adolescents, TMD was more prevalent among girls than boys, with a lower prevalence than in adults^14^. TMD in adolescents can lead to permanent complications involving joint damage or deficits in mandibular growth, resulting in micrognathia, posterior rotation of the mandible, and malocclusion^16^. However, significant knowledge gaps remain in case of adolescent TMD.

TMD pain in adolescents may be multifactorial, involving a complex growth trajectory from the biopsychosocial model^17^. Older girls aged between 16 and 19 years had significantly higher pain scores than did younger boys aged between 12 and 15 years^18^, as well as higher analgesic consumption and school absences than did older boys^19^. In addition, highly anxious adolescents tend to function poorly, regardless of the level of pain^20^. That is, the pain experience has different aspects depending on the sex, and it may change or be affected by the psychological state. Somatic complaints and headache have been strongly associated with TMD pain in a previous study of a population-based sample of 12- to 19-year-olds^10^. In previous studies on adult patients with TMD, anxiety correlated with clinical signs of TMD and muscle tenderness^21^. With regard to a macrotrauma history, patients with whiplash trauma had higher scores of muscle pain and psychological distress, as well as a poorer prognosis, than did those without a whiplash history^22^. According to a review of the literature, adolescents with traumatic experiences had comorbid conditions, including anxiety, sleep problems, and attention and learning problems^23^. Although macrotrauma plays an important role in the development, maintenance, and worsening of TMD symptoms, it has rarely been investigated in adolescents with TMD.

Magnetic resonance imaging (MRI) is considered the gold standard for evaluating physical and/or structural abnormalities of the TMJ and adjacent structures in patients with TMD. MRI provides high tissue contrast, while being noninvasive and radiation-free^24^. In previous MRI studies, anterior disc displacement was most prevalent in adolescents with TMD, while bone changes were more prevalent in the elderly^25,26^. Disc displacement was reported to precede disc degeneration, joint effusion, and degenerative osseous changes of the condyle and temporal bone^24^. However, few MRI studies have been performed in adolescents with TMD. Moreover, findings regarding the effects of trauma on the symptoms and signs of TMD in the young have been inconsistent, and no comparison has been made between the MRI findings of patients with TMD, with or without trauma.

Therefore, the aim of the present study was to evaluate the clinical, MRI, and psychological characteristics of TMD signs and symptoms, and their relationships in adolescents, as well as to compare these findings between young and older adolescent patients, in the context of the relevant available literature. Additionally, we aimed to determine whether psychological impairments generally considered predictive of TMD are associated with increased pain intensity and a history of trauma.

## Methods

### Patient selection

We retrospectively analyzed the data of patients aged between 12 and 19 years who visited the orofacial pain clinic at Kyung Hee University Dental Hospital between January 2013 and January 2019 because of TMD symptoms. They were diagnosed with TMD according to the RDC/TMD^6^ and underwent bilateral TMJ-MRI, including closed- and open-mouth views, during the first visit.

Among them, 35 patients were randomly selected, using a simple random sampling procedure employing a random number table for each of two age groups: those aged 12–16 years were designated as young adolescents, and those aged 17–19 years were designated as older adolescents. Thus, the present study included 70 adolescents with TMD (31 female and 39 male patients; mean age: 16.46 ± 2.36 years). Patients in both groups were sub-divided according to the presence (T1) or absence (T0) of a trauma history and were further analyzed. The exclusion criteria were as follows: subjects with a history of facial fracture injury, ongoing orthodontic treatment that could interfere with osteoarthritis, systemic osteoarthritis, and those with juvenile idiopathic arthritis. Written informed consent was obtained from all study patients. In patients under the age of 18 years, informed consent was obtained from a parent and/or legal guardian. The study design was approved by the appropriate ethics review boards of Kyung Hee University Dental Hospital.

### MRI acquisition and analysis

TMJ-MRI was performed using a 1.5 T MRI system (Signa Genesis; GE Healthcare, Chicago, IL, USA) employing a 6 × 8-cm-diameter surface coil. The protocol for TMJ examination included T2-weighted imaging (T2WI), T1-weighted imaging (T1WI), and proton density (PD) imaging of both TMJs in the coronal and sagittal oblique planes by using thin sections of 3 mm or less, with a 15 cm field of view and a 256 × 224 matrix. T2W, T1W, and PD images were acquired at 2650/82, 650/14, and 2650/82 repetition time/echo time sequences. Two experienced head and neck radiologists, blinded to the patients’ clinical information, performed visual analyses of the MR images. An initial analysis was first carried out to estimate the agreement between the two radiologist’s opinions by using dichotomous levels to reflect a clinically significant change with Kappa statistics. The Kappa coefficient was 0.93, indicating a high level of agreement between the two radiologists. After the TMJ images were acquired, the TMJ was evaluated using an Infinitt Picture Archiving and Communication System (Infinitt Corp., Seoul, Korea). Images of the TMJ in the sagittal and coronal planes were acquired to determine the presence of internal TMJ derangement, including disc displacement with and without reduction, effusion, disc deformity, and condylar degeneration. Disc position was determined in the closed- and open-mouth positions in the oblique sagittal plane.

### Assessment

A clinical examination was performed in accordance with the RDC/TMD, and maximum unassisted pain-free jaw opening as well as mandibular movement capacity and associated pain were measured in millimeters, with a ruler, between the maxillary and mandibular central incisors. The presence of joint sounds and palpatory pain of the temporomandibular muscles and joints were also assessed. Thus, the following multiple diagnoses could be made: myofascial pain, disc displacement, and/or arthralgia/osteoarthrosis. Two examiners (L.Y.H. and H.J.P.), trained and specialized in orofacial pain and TMD, with over 5 years of experience, were calibrated for diagnosing TMD based on the RDC/TMD criteria^6^. The Kappa coefficient for inter-examiner diagnostic agreement was 0.95. The acceptable reliability of the questionnaire, clinical examination, and diagnosis has been previously reported^27^. In the questionnaire, the patients reported the intensity, frequency, duration, and location of TMD-related symptoms and jaw function. In terms of the factors contributing to TMD, the patients were asked if they had been told or whether they themselves had noticed that they had each contributing factor, and their responses were recorded as “yes” or “no.”

The T1 subgroup comprised patients with TMD in whom TMD symptoms occurred after macrotrauma to their neck and facial areas. The types of trauma included violent attacks (n = 10), car accidents (n = 6), falling-down injury (n = 3), and being hit by something (n = 11). Psychological characteristics were evaluated using the Symptom Checklist-90-Revised (SCL-90-R). The SCL-90-R comprises nine symptom subscales, including somatization, obsessive-compulsiveness, interpersonal sensitivity, depression, anxiety, hostility, phobic anxiety, paranoid ideation, and psychosis, and three global indices of functioning, including the global severity index (GSI), positive symptom distress index, and positive symptom total (PST).

### Clinical and MRI Data collection

All patients completed questionnaires regarding TMD pain and dysfunction, and all were assessed according to RDC/TMD Axis I^27^. The adolescents rated their subjective TMD-related pain intensity on a 0–10 visual analogue scale (VAS). The severity of TMD was measured using TMD indexes, including a palpation index (PI), dysfunction index (DI), and craniomandibular index (CMI)^28^. In addition, mandibular movement in the centric position (CMO, comfortable mouth opening without pain; and MMO, unassisted maximum mouth opening with pain) and eccentric positions (protrusion and lateral movement) were investigated.

**Figure 1:**
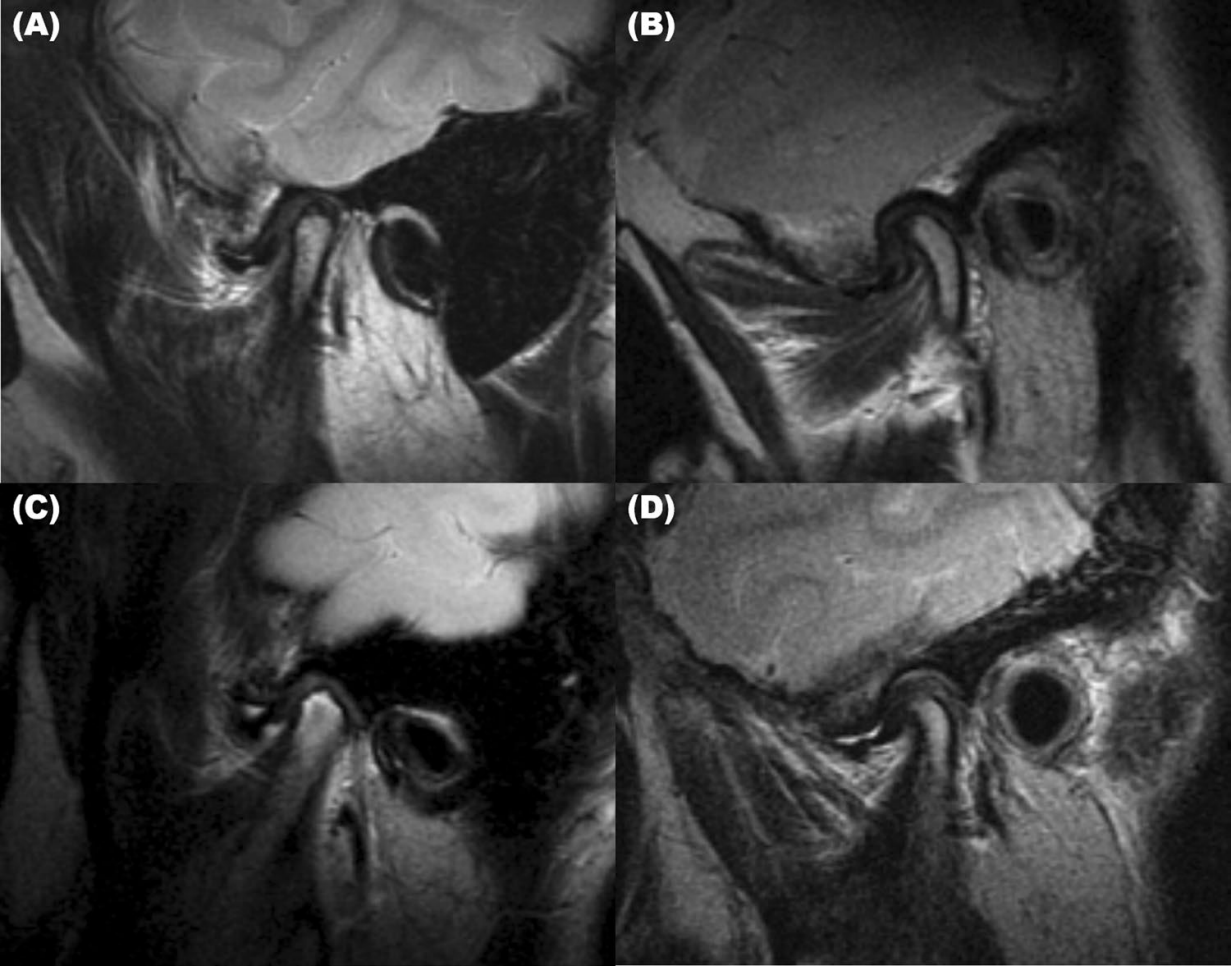
Magnetic resonance imaging (MRI) features of the abnormal temporomandibular joint disc and mandibular condyle. (**A**) Anterior disc displacement. Sagittal oblique T2-weighted image (T2WI) acquired in the closed-mouth position shows an anteriorly displaced disc. (**B**) Disc deformity. Sagittal T2WI shows a dysmorphic and anteriorly displaced disc. (**C**) Condylar degeneration. T2WI shows arthritis of the mandibular condyle. (**D**) Effusion. T2WI shows clearly delineated articular fluid collection with hyperintensity, degeneration of the mandibular condyle, and an anteriorly displaced disc.

We used the MRI findings to investigate the presence of disc displacement (Fig. 1A), disc deformity (Fig. 1B), condylar degeneration (Fig. 1C), and effusion (Fig. 1D). To determine whether differences between the measurements were statistically significant, masticatory muscle values in each MR sequence of the right and left sides of the same patients were compared.

### Statistical methods

Descriptive statistics were used to present percentages, means, and standard deviations (SDs) for continuous variables. Student’s t-test, for non-normally distributed variables, and chi-square test, for normally distributed variables, were used to compare the young and older adolescents, and patients with trauma vs. patients without trauma in each adolescent group. Differences in the means of continuous variables between the independent groups were examined using Student’s t-test. Fisher’s exact test was used to determine the equality of proportions. For analyzing the bivariate correlations between categorical and continuous variables, the chi-square test and Pearson’s correlation test were used. The Kappa statistics was used to measure the agreement degree (Kappa coefficient) between the two examiners who evaluated and rated the same subjects.

Logistic regression analysis was performed, with each psychological factor as a dependent variable and the presence of a trauma history as an independent variable. The regression coefficient value (B), standard error (SE), p-values, and odds ratios (ORs) with 95% confidence intervals (95% CIs) were investigated. Spearman’s correlation analysis was performed to investigate the relationship between psychological factors and the TMD indexes. A two-tailed p-value < 0.05 was considered statistically significant. Data were analyzed using IBM SPSS Statistics for Windows, Version 20.0 (IBM Corp., Armonk, NY, USA).

### Ethics approval and consent to participate

Procedures on human subjects were performed in accordance with the ethical standards of the Committee on Human Experimentation of our institution as well as the Helsinki Declaration of 1975. In addition, the study design was approved by the appropriate ethics review boards of Kyung Hee University Dental Hospital.

## Results

### Demographic characteristics of patients and pain intensity

Patients’ characteristics are given in Table 1. The mean age of young adolescents was 14.54 ± 1.80 years, and that of older adolescents was 18.37 ± 0.69 years. The symptom duration of young adolescents was 149.63 ± 217.24 days (4.1 ± 7.6 months), which was significantly shorter than that of older adolescents 467.23 ± 500.28 days (15.6 ± 16.7 months) (p = 0.001). The ratio of girls to boys was significantly higher among young adolescents (65.7%) than among older adolescents (22.9%) (p = 0.006). The distribution of symptoms reported by the patients, the side affected by osteoarthritis, and the prevalence rate of osteoarthritis were not significantly different between the two TMD groups. TMJ pain was the most prevalent symptom among young adolescents, while TMJ noise was the most prevalent among older adolescents. In terms of the range of mandibular movements, the mean CMO and of MMO values were significantly smaller among young adolescents than among older adolescents. The mouth opening range usually increases with age until 14 years of age and peaks in individuals between 14 and 30 years of age^29,30^. Although reduced mouth opening has been known to have diagnostic value for assessing the TMD status and the function of the TMJ, reports of measuring the mouth opening range in adolescents with TMD are lacking. As compared to our previous results^31^, it is reasonable to assume that these levels are affected by both TMD status and body growth, as only the CMO value is smaller in young adolescents than in adults with TMD (32.31 ± 10.42 vs. 34.56 ± 11.20 mm, p = 0.003).

**Table 1:** Characteristics of the patients.

Group	Young adolescents (n = 35)	Older adolescents (n = 35)	P-value
Mean age (year)^†^	**14.54** ± **1.80**	**18.37** ± **0.69**	0.000*
Girls, n (%)^‡^	**23 (65.7%)**	**8 (22.9%)**	0.006**
Symptom duration^†^	**149.63** ± **217.24**	**467.23** ± **500.28**	0.001*
**Clinical diagnosis**			
Arthralgia by RDC/TMD^‡^			
Rt. side	8 (22.9%)	12 (34.3%)	0.392
Lt. side	4 (36.4%)	7 (63.6%)	
Both sides	17 (48.6%)	11 (31.4%)	
Osteoarthrosis by RDC/TMD^‡^	6 (17.1%)	5 (14.3%)	0.500
**Reported symptom**^**‡**^			
TMJ noise	26 (74.3%)	29 (82.9%)	0.281
TMJ pain	32 (91.4%)	28 (80.0%)	0.153
Stiffness	11 (31.4%)	10 (28.6%)	0.500
Locking	9 (25.7%)	16 (45.7%)	0.067
**Range of mandibular movement**			
CMO^†^	**32.31** ± **10.42**	**37.29** ± **10.14**	0.047*
MMO^†^	**41.26** ± **10.31**	**46.69** ± **10.43**	0.032*
Subjective pain intensity			
VAS^†^	5.12 ± 2.45	4.96 ± 2.74	0.794
TMD indexes			
DI^†^	0.361 ± 0.242	0.425 ± 0.287	0.316
PI^†^	**0.461** ± **0.354**	**0.275** ± **0.237**	0.012*
CMI^†^	0.411 ± 0.252	0.350 ± 0.229	0.295

^†^Mann-Whitney U test was used to determine significant mean differences between the 2 TMD groups.

^‡^Fisher’s exact test was used to determine whether there was a difference in clinical distribution between the 2 TMD groups.

P-value significance was set at <0.05. *p-value < 0.05, **p-value < 0.01.

Significant variables and results are shown in bold text.

CMO: comfortable mouth opening, MMO: maximum mouth opening, DI: dysfunction index, CMI:

craniomandibular index, VAS: visual analogue scale.

The VAS score, which shows the degree of subjective pain, was higher in young adolescents, but the difference was not significant (p = 0.794). The PI, which shows the severity of muscle pain, was significantly higher among young adolescents than older adolescents (p = 0.012).

### Distribution of clinical symptoms and contributing factors

The distribution of TMD symptoms and contributing factors was significantly different between the young and older adolescents (Table 2). The presence of tinnitus (8.6% vs. 28.6%), headache (40.0% vs. 60.0%), history of maxillary orthodontic treatment (0.0% vs. 22.9%), and sleep problems (0.0% vs. 14.3%) was significantly lower among young adolescents than among older adolescents. Conversely, the prevalence of a preference for hard food (37.1% vs. 14.3%), excessive talking (37.1% vs. 11.4%), and a history of trauma (54.3% vs. 31.4%) was significantly higher among young adolescents than among older adolescents. The most prevalent contributing factor in young adolescents was a history of trauma, and that among older adolescents was unilateral chewing (34.3%).

**Table 2:** Distribution of comorbid conditions and contributing factors, as well as a comparison between the temporomandibular disorder (TMD) groups.

	Young adolescents (n = 35)		Older Adolescents (n = 35)		P-value
	Frequency	(%)	Frequency	(%)	
**Comorbid conditions**^**‡**^					
**Tinnitus**	**3**	**(8.6%)**	**10**	**(28.6%)**	0.031*
**Headache**	**14**	**(40.0%)**	**22**	**(62.9%)**	0.047*
**Orthodontic treatment Hx**.	**0**	**(0.0%)**	**8**	**(22.9%)**	0.002**
Abnormal occlusion	5	(14.3%)	6	(17.1%)	0.500
Stress	17	(48.6%)	15	(42.9%)	0.405
Family Hx.	2	(5.7%)	0	(0.0%)	0.246
**Contributing factors**^**‡**^					
Bruxism	4	(11.4%)	4	(11.4%)	1.000
Clenching	11	(31.4%)	7	(20.0%)	0.206
Perioral contraction	4	(11.4%)	2	(5.7%)	0.337
Tongue thrusting	0	(0.0%)	0	(0.0%)	
Lip and nail biting	13	(37.1%)	8	(22.9%)	0.148
Chin buttressing	11	(31.4%)	6	(17.1%)	0.132
Unilateral chewing	12	(34.3%)	12	(34.3%)	1.000
**Hard food**	**13**	**(37.1%)**	**5**	**(14.3%)**	0.027*
Irregular diet	5	(14.3%)	6	(17.1%)	1.000
Indigestion	9	(25.7%)	6	(17.1%)	0.281
Coffee	2	(5.7%)	4	(11.4%)	0.337
**Sleep problem**	**0**	**(0.0%)**	**5**	**(14.3%)**	0.027*
High pillow	1	(2.9%)	2	(5.7%)	1.000
Unilateral sleep	12	(34.3%)	11	(31.4%)	1.000
Bad posture	17	(48.6%)	13	(37.1%)	0.235
**Much talking**	**13**	**(37.1%)**	**4**	**(11.4%)**	0.012*
Cold weather	2	(5.7%)	1	(2.9%)	1.000
**Trauma Hx**.	**19**	**(54.3%)**	**11**	**(31.4%)**	0.045*

^‡^Fisher’s exact test was used to determine whether there was a difference in clinical distribution between the 2 TMD groups.

P-value significance was set at <0.05. *p-value < 0.05, **p-value < 0.01, ***p-value < 0.001.

Significant variables and results shown in bold text.

TMJ: temporomandibular joint, Hx.: history.

### Distribution of mri findings according to the adolescent groups

We investigated the presence of disc displacement, disc deformity, condylar degeneration, and effusion on MR images of both TMJs (Table 3). Only anterior displacement, and not posterior displacement of the TMJ disc, was observed. Anterior disc displacement was observed at a substantial rate among both young and older adolescents. The prevalence of disc displacement (80% vs. 54.3%) and effusion (60% vs. 28.6%) of the right TMJ was significantly higher among young adolescents than older adolescents. The most frequently observed MRI findings did not differ between the age groups; that is, in both young and older adolescents, disc displacement was the most common MRI finding, followed by disc deformity, effusion, and condylar degeneration. Distribution of these MRI findings on the left side was not significantly different between the groups. Interestingly, when we analyzed the data according to the presence or absence of a history of trauma, no significant differences were observed in the MRI variables in either of the adolescent groups.

**Table 3:** Comparison of the prevalence of abnormal findings on magnetic resonance imaging (MRI).

	Young adolescents	Older Adolescents	P-value	Young adolescents		P-value	Older Adolescents		P-value
	Total (n = 35)	Total (n = 35)		Non-trauma^§^ (n = 16)	Trauma^¶^ (n = 19)		Non-trauma^§^ (n = 24)	Trauma^¶^ (n = 11)	
**Rt. Side**^**‡**^									
**Disc displacement**	**28 (80.0%)**	**19 (54.3%)**	0.020*	12 (34.3%)	16 (45.7%)	0.623	12 (34.3%)	7 (20.0%)	0.352
ADD with reduction	9 (25.7%)	8 (22.9%)		3 (8.6%)	6 (17.1%)	0.398	3 (8.6%)	5 (14.3%)	0.071
ADD without reduction	19 (63.3%)	11 (31.4%)		9 (25.7%)	10 (28.6%)		9 (25.7%)	2 (5.7%)	
Disc deformity	20 (57.1%)	17 (48.6%)	0.632	10 (28.6%)	10 (28.6%)	0.734	11 (31.4%)	6 (17.1%)	0.725
Condylar degeneration	13 (37.1%)	13 (37.1%)	1.000	7 (20.0%)	6 (17.1%)	0.347	10 (28.6%)	3 (8.6%)	0.334
**Effusion**	**21 (60.0%)**	**10 (28.6%)**	0.015*	9 (25.7%)	12 (34.3%)	0.739	6 (17.1%)	4 (11.4%)	0.380
**Lt. side**^**‡**^									
Disc displacement	19 (54.3%)	21 (60.0%)	0.809	9 (25.7%)	10 (28.6%)	0.690	12 (34.3%)	2 (5.7%)	0.077
ADD with reduction	6 (17.1%)	10 (28.6%)		2 (5.7%)	4 (11.4%)	1.000	4 (11.4%)	6 (17.1%)	0.055
ADD without reduction	13 (37.1%)	11 (31.4%)		7 (20.0%)	6 (17.1%)		8 (22.9%)	3 (8.6%)	
Disc deformity	17 (48.6%)	20 (57.1%)	0.632	7 (20.0%)	10 (28.6%)	0.427	12 (34.3%)	8 (22.9%)	0.187
Condylar degeneration	10 (28.6%)	13 (37.1%)	0.611	4 (11.4%)	6 (17.1%)	0.481	10 (28.6%)	3 (8.6%)	0.334
Effusion	18 (51.4%)	17 (48.6%)	1.000	8 (22.9%)	10 (28.6%)	0.573	10 (28.6%)	7 (20.0%)	0.289

^‡^Fisher’s exact test was used to determine whether there was a difference in distribution of clinical factor between the two groups. ADD: anterior disc displacement, Rt.: right, Lt.: left.

P-value significance was set at <0.05. *p-value < 0.05. Significant variables and results shown in bold text.

### Psychological factors and trauma history

No significant difference was observed in the SCL-90-R subscales between young and older adolescents. The mean T-score of somatization was the highest among the nine psychological subscales.

In young adolescents with TMD, a significant difference was observed in the T-scores of the SCL-90-R subscales between those with (T1) and those without (T0) a history of trauma. The T1 subgroup had significantly higher T-scores in five of the symptom subscales of the SCL-90-R, including somatization (50.5 vs. 41.9), obsessive-compulsivity (45.5 vs. 37.9), hostility (46.3 vs. 41.7), phobic ideation (49.4 vs. 42.9), and psychosis (47.7 vs. 42.3) than did the T0 subgroup. The mean difference in the anxiety T-score between the T0 and T1 subgroups (45.0 vs. 40.3) was borderline significant (p = 0.063). Furthermore, among young adolescents, the T1 subgroup presented with higher global indices of GSI and PST than did the T0 subgroup. In contrast, among older adolescents, no significant differences were observed in the SCL-90-R subscales between T0 and T1.

### Logistic regression analysis results

**Figure 2:**
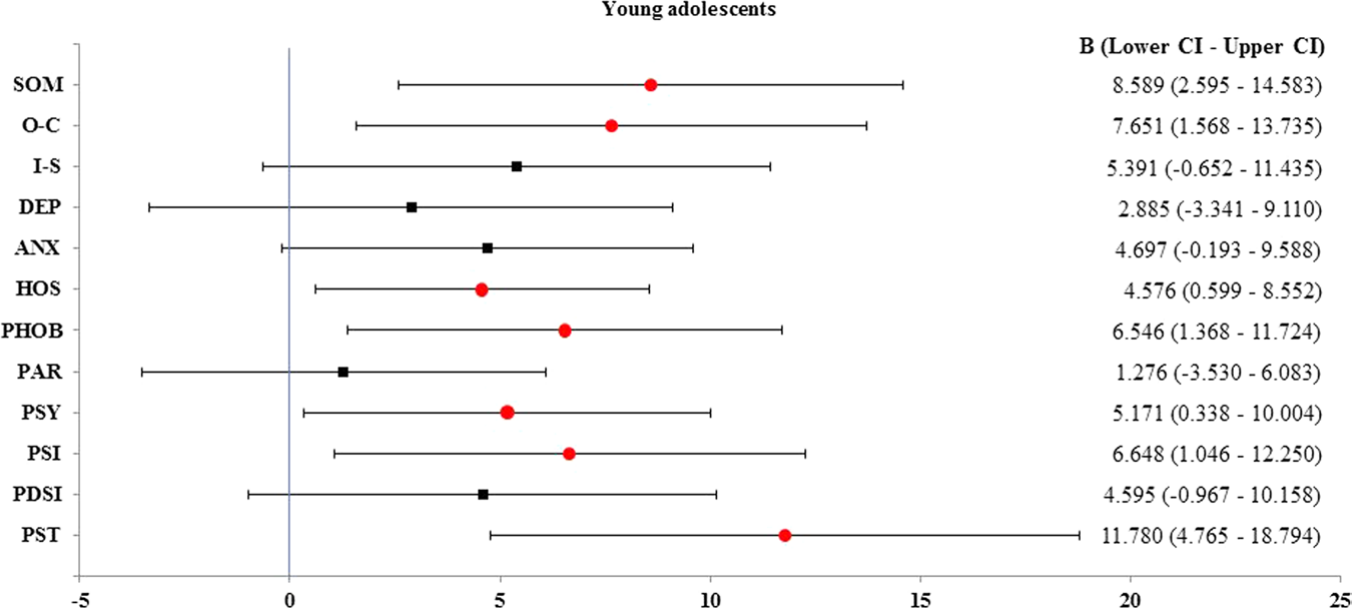
Linear regression analysis in young adolescents. From the linear logistic regression analysis, the beta coefficient is the degree of change in the outcome variable (dependent variable) for every 1 unit of change in the predictor variable (independent variable). The circle and square show the means of the beta coefficient. If zero (0) is included in the beta coefficient range, it is not a significant result. Thus, the circle shows that the average value of the beta coefficient increases significantly when trauma is present, and the square shows that the increase is not significant. SOM: somatization, O-C: obsessive-compulsiveness, I-S: interpersonal sensitivity, DEP: depression, ANX: anxiety, HOS: hostility, PHOB: phobic anxiety, PAR: paranoid ideation, PSY: psychosis, B: beta coefficient, CI: confidence interval.

**Figure 3:**
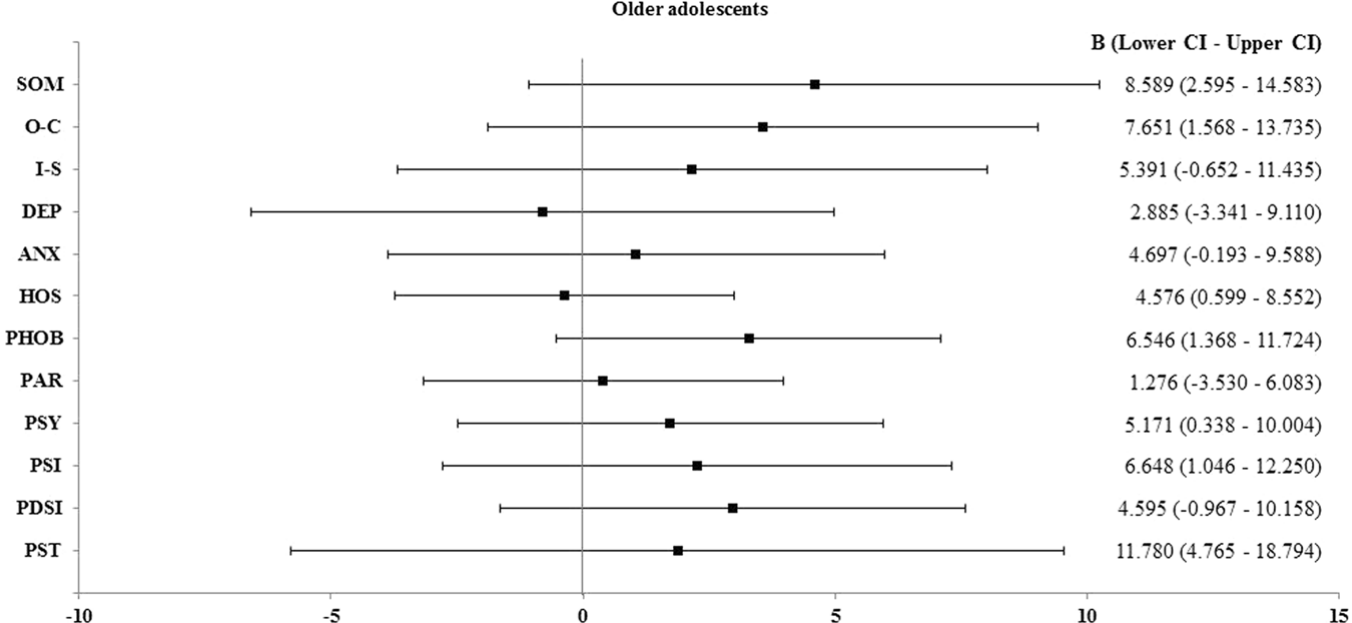
Linear regression analysis in older adolescents. From the linear logistic regression analysis, the beta coefficient is the degree of change in the outcome variable (dependent variable) for every 1 unit of change in the predictor variable (independent variable). The square shows the means of the beta coefficient. When zero (0) is included in the beta coefficient range, the result is not significant. Thus, the mean beta coefficients obtained from all psychological variables are not associated with the presence of a history of trauma in older adolescents. SOM: somatization, O-C: obsessive-compulsiveness, I-S: interpersonal sensitivity, DEP: depression, ANX: anxiety, HOS: hostility, PHOB: phobic anxiety, PAR: paranoid ideation, PSY: psychosis, B: beta coefficient, CI: confidence interval.

Logistic regression analysis yielded noteworthy results only among young adolescents. The presence of a trauma history statistically significantly increased the scores of six subscales: somatization, obsessive-compulsiveness, anxiety, hostility, phobic ideation, and psychosis, by 8.589, 7.651, 4.697, 4.576, 6.546, and 5.171, respectively (Fig. 2). This was consistent with the results shown in Table 4, and a history of trauma resulted in the greatest increase in the logistic regression coefficient value for somatization. In contrast, the presence of trauma did not significantly increase the score of any of the SCL-90-R subscales among older adolescents (Fig. 3).

**Table 4:** Comparisons of psychological factors according to a history of trauma.

	Young adolescents	Older adolescents	P-value	Young adolescents		P-value	Older adolescents		P-value
	Total (n = 35)	Total (n = 35)		Non-trauma^§^ (n = 16)	Trauma^¶^ (n = 19)		Non-trauma^§^ (n = 24)	Trauma^¶^ (n = 11)	
	mean ± SD	mean ± SD		mean ± SD	mean ± SD		mean ± SD	mean ± SD	
**SOM**	46.60 ± 9.59	43.4 ± 7.84	0.131	**41.94 ± 8.01**	**50.53 ± 9.20**	0.006**	41.96 ± 7.35	46.55 ± 8.29	0.133
**O-C**	42.03 ± 9.50	39.91 ± 7.45	0.304	**37.88 ± 9.94**	**45.53 ± 7.75**	0.018*	38.79 ± 6.27	42.36 ± 9.43	0.271
I-S	43.11 ± 9.04	40.43 ± 7.85	0.189	40.19 ± 11.64	45.58 ± 5.25	0.102	39.75 ± 7.81	41.91 ± 8.10	0.468
DEP	41.63 ± 9.00	40.91 ± 7.70	0.722	40.06 ± 9.28	42.95 ± 8.79	0.355	41.17 ± 7.49	40.36 ± 8.50	0.791
ANX	42.80 ± 7.37	41.91 ± 6.58	0.598	40.25 ± 7.65	44.95 ± 6.58	0.063	41.58 ± 5.64	42.64 ± 8.56	0.715
HOS	44.17 ± 6.13	43.34 ± 4.47	0.520	**41.69** ± **6.41**	**46.26** ± **5.16**	0.029*	43.46 ± 4.37	43.09 ± 4.89	0.834
**PHOB**	46.43 ± 8.10	44.20 ± 5.30	0.178	**42.88** ± **7.82**	**49.42** ± **7.22**	0.016*	43.17 ± 4.68	46.45 ± 6.09	0.132
PAR	41.94 ± 6.89	39.54 ± 4.75	0.094	41.25 ± 9.30	42.53 ± 4.10	0.616	39.42 ± 5.38	39.82 ± 3.16	0.784
**PSY**	45.06 ± 7.38	42.54 ± 5.66	0.114	**42.25** ± **7.06**	**47.42** ± **6.95**	0.037*	42.00 ± 5.21	43.73 ± 6.66	0.459
**GSI**	42.17 ± 8.67	40.09 ± 6.80	0.267	**38.56** ± **9.22**	**45.21** ± **7.06**	0.026*	39.38 ± 6.29	41.64 ± 7.90	0.416
PDSI	44.06 ± 8.27	41.69 ± 6.30	0.182	41.56 ± 10.28	46.16 ± 5.56	0.123	40.75 ± 6.52	43.73 ± 5.50	0.175
**PST**	40.46 ± 11.52	38.17 ± 10.12	0.079	**34.06** ± **10.14**	**45.84** ± **9.77**	0.000***	37.58 ± 9.24	39.45 ± 11.83	0.312

Correlation was analyzed via Spearman’s correlation analysis.

P-value significance was set at <0.05. *p-value < 0.05. **p-value < 0.01. ***p-value < 0.001. Significant variables and results shown in bold text.

SOM: somatization, O-C: obsessive-compulsive, I-S: interpersonal sensitivity, DEP: depression, ANX: anxiety, HOS: hostility, PHOB: phobic anxiety,

PAR: paranoid ideation, PSY: psychosis, SD: standard deviation.

Non-trauma: ^§^Patients who had non-traumatic TMD symptoms without any history of head/neck trauma.

Trauma: ^¶^Patients who had post-traumatic TMD symptoms and had no TMD symptoms before the trauma.

### Correlations between the TMD indexes and SCL-90-R subscales

Overall, the scores of the TMD indexes and T-scores of the SCL-90-R subscales were positively correlated (Table 5). The specific correlations of each TMD group and subgroup showed some similarities and differences. The PI was positively correlated with somatization in both the TMD groups and their subgroups. In the T1 subgroup of both the TMD groups, the PI displayed a significant positive correlation with hostility; the DI showed a positive correlation with somatization, obsessive-compulsiveness, anxiety, psychosis, and the three global indices; and the CMI displayed a positive correlation with obsessive-compulsiveness. However, conflicting results were observed in the T0 subgroups regarding the relationship between the PI and anxiety and between the CMI and depression; these were positively correlated in the T0 subgroup of young adolescents, whereas these were negatively correlated in the T0 subgroup of older adolescents.

**Table 5:** Correlation between the Symptom Checklist-90-Revised subscales and the temporomandibular disorder (TMD) indexes in young vs. older adolescents.

	Young adolescents								Older adolescents							
	Non-trauma^§^ (n = 16)				Trauma^¶^ (n = 19)				Non-trauma^§^ (n = 24)				Trauma^¶^ (n = 11)			
	VAS	PI	DI	CMI	VAS	PI	DI	CMI	VAS	PI	DI	CMI	VAS	PI	DI	CMI
SOM	−0.042	0.253*	0.231	0.188	−0.299**	0.250*	0.318**	0.208	−0.113	0.210*	0.006	0.151	0.179	0.507**	0.448**	0.477**
O-C	0.215	0.084	0.050	0.042	−0.034	0.261*	0.405**	0.280*	−0.046	0.038	−0.036	−0.078	0.327*	0.263	0.334*	0.335*
I-S	0.135	0.145	0.002	0.052	−0.222	0.008	0.299**	0.078	0.015	0.000	−0.138	−0.074	0.465**	0.246	0.234	0.222
DEP	0.295*	0.331**	0.176	0.252*	−0.219	0.117	0.045	−0.019	0.063	−0.191	−0.066	−0.258*	0.430**	0.192	0.342*	0.281
ANX	0.192	0.270*	0.266*	0.237	−0.339**	0.322**	0.313**	0.248*	0.058	−0.220*	−0.137	−0.297**	0.535**	0.275	0.342*	0.271
HOS	0.422**	0.121	0.197	0.144	−0.184	0.240*	0.124	0.102	−0.035	−0.149	−0.109	−0.288**	0.136	0.337*	0.158	0.238
PHOB	−0.040	0.120	0.277*	0.184	−0.−0.180	0.155	0.292*	0.142	−0.224*	−0.322**	0.070	−0.264**	0.248	0.275	0.239	0.271
PAR	−0.089	0.026	−0.220	−0.147	−0.276*	0.174	0.078	0.028	−0.143	0.157	−0.083	0.106	0.204	0.219	−0.017	0.075
PSY	0.332**	0.337**	0.263*	0.296*	−0.205	0.113	0.260*	0.096	0.186	−0.117	−0.154	−0.193	0.256	0.315*	0.346*	0.363*
GSI	0.150	0.198	0.100	0.115	−0.284*	0.298**	0.332**	0.227*	0.146	−0.018	−0.117	−0.124	0.340*	0.254	0.311*	0.296
PDSI	0.023	0.362*^*^	−0.003	0.180	−0.287*	0.287*	0.388**	0.279*	−0.333**	0.008	0.166	0.035	0.298*	0.233	0.342*	0.340*
PST	0.219	0.125	0.036	0.050	−0.187	0.333**	0.438**	0.309^*^*	0.075	−0.007	−0.119	−0.139	0.260	0.275	0.342*	0.345*

Correlation was analyzed via Spearman’s correlation analysis.

P-value significance was set at <0.05. *p-value < 0.05. **p-value < 0.01. Significant variables and results shown in bold text.

SOM: somatization, O-C: obsessive-compulsive, I-S: interpersonal sensitivity, DEP: depression, ANX: anxiety, HOS: hostility, PHOB: phobic anxiety, PAR: paranoid ideation, PSY: psychosis, SD: standard deviation

Non-trauma: ^§^Patients who had non-traumatic TMD symptoms without any history of head/neck trauma. Trauma: ^¶^Patients who had post-traumatic TMD symptoms and had no TMD symptoms before the trauma.

Among young adolescents, additional significant correlations were found. In the T0 subgroup of young adolescents, the PI was positively correlated with depression and psychosis, but this was not observed in the T0 subgroup of older adolescents. In the T1 subgroup of young adolescents, the PI was positively correlated with obsessive-compulsiveness and anxiety, but this was not observed in the T1 subgroup of older adolescents.

## Discussion

To our knowledge, no previous study has comprehensively investigated the relationship between a history of macrotrauma and clinical TMD symptoms, MRI findings, and psychological factors in adolescents with TMD. Interestingly, the TMD in young adolescents is unique, and clinicians should be more concerned about increased muscle pain and the relationship between psychological characteristics and a history of trauma. Although patients aged younger than 18 years have been commonly excluded from TMD studies^32^, TMD research in adolescents is indispensable. Approximately 4% of adolescents aged 12–19 years had TMD pain, 8–38% had headache, and 4–40% had musculoskeletal pain^18,33^. The lack of an identifiable etiology along with the complex biopsychosocial nature of adolescent TMD leads to delayed treatment that can exacerbate existing symptoms. Therefore, the results of this study provide clinical and imaging characteristics of adolescents with TMD and the use of these characteristics in making treatment decisions.

We emphasize that the two age-divided adolescent TMD groups have different clinical characteristics. First, this study showed that young adolescents with TMD presented higher PI scores (0.461 ± 0.354 vs. 0.275 ± 0.237) and a higher prevalence of a history of trauma (54.3% vs. 31.4%) than older adolescents. According to Kim *et al*., the PI scores increase significantly with a history of trauma in patients with TMD^34^. TMD pain was reported to increase with increasing age in adolescents aged 12–19 years^18^, which was consistent with our results. Nilsson *et al*. analyzed the overall level of TMD pain, whereas we investigated muscle- and joint-origin pain. As the PI measures the level of muscle tenderness in the stomatognathic system, this index separates joint problems from muscle problems^28^. Conversely, older adolescents with TMD presented with a higher prevalence of headache (40.0% vs. 62.9%). Headaches appear to be strongly associated with TMD in adolescents, with headaches most commonly accompanied by TMD pain^10^. Additional studies are needed to clarify the relationship between headache and aging in adolescents with TMD.

Young adolescents with TMD presented a shorter symptom duration than did older adolescents (149.63 ± 217.24 days vs. 467.23 ± 500.28 days). The role of parental influence in the significantly shorter symptom duration among young adolescents may be considered, because they visited accompanied by parents at a higher frequency than did older adolescents. Parental influence is relatively more important in developmental changes during early adolescence; thereafter, it slowly decreases, as peer influence increases throughout adolescence^35^. Parental influence can affect both the child’s self-esteem and self-efficacy, as well as pain perception, pain experience, and pain behavior^36^. With regard to the presence of parafunctional habits and contributing factors, significant differences were observed between the adolescent TMD groups. Parafunctional habits, including bruxism, clenching, and other repetitive habitual behaviors are associated with psychological distress and are thought to contribute to the development of TMD via joint overload, which leads to cartilage breakdown, synovial fluid changes, and other changes within the joint^2,37^. However, the paucity of research on adolescent TMD is too great to conclude that a relationship exists between parafunctional habits and TMD. Taken together, we hypothesized that the etiology of the two adolescent TMD groups would be different.

Regarding the presence of a trauma history on MRI analysis, our results showed that the prevalence of TMJ abnormalities was not different between the groups; this may imply a weak correlation between a history of trauma and structural changes to the TMJ in adolescents with TMD. Three MRI studies performed on patients who developed TMJ symptoms after macrotrauma, such as whiplash injury, revealed the presence of disc displacement in 56%^38^, 87%^39^, and 40%^40^ of the joints. In each study the prevalence of disc displacement was higher than that in the control group without trauma. However, in one previous prospective study the prevalence of disc displacement on MRI did not differ significantly between patients with and without whiplash trauma at either of the two follow-up MRI examinations at 1 year and 15 years^41^.

We also found that anterior disc displacement was a prominent MRI finding in both age-divided subgroups; moreover, all disc displacement was anterior. Vogl *et al*. reported that anterior disc displacement was observed in 35% of adults clinically diagnosed with TMD and posterior disc displacement was observed in only 3%^24^. According to Su *et al*., disc changes were more prevalent in adolescents with TMD than in older patients with TMD, and disc displacement was the most common finding^25^, which is consistent with our results. Joint effusion was observed in 44.2% of all adolescents with TMD and in 53.3% of individuals with microtrauma. The prevalence of joint effusion varied in previous studies; Pressman *et al*. reported effusion in 65% of the joints in patients with TMD and macrotrauma^38^, whereas others reported this in only 6% of TMJs in the whiplash trauma patient group^40^. In our study no significant difference was observed in the MRI findings according to age or a macrotrauma history. Knowledge regarding the relationship between macrotrauma and physical changes observed on MRI in adolescents with TMD has remained limited and few studies have been conducted to allow us to draw clear conclusions.

Nevertheless, the proximate relationship between the presence of trauma and aggravation of psychological factors was clear. It was noteworthy that somatization, obsessive-compulsiveness, hostility, phobic ideation, psychosis scores, and GSI were significantly higher in young adolescents with a history of trauma. That is, unlike in older adolescents, a macrotrauma history increases psychological distress in young adolescents. The development of autonomy from parents is an important developmental task for adolescents^42^. Sense of autonomy increased across ages 13 to 19 and rose sharply between ages 15 and 17^43^, indicating that steep increases occur in late adolescence. In addition, impaired autonomy is related to chronic pain, pain-related disability, and psychological distress^44,45^. These findings may be the basis for explaining how TMD clinical characteristics differ according to the age of adolescents. Patients with TMD and a history of trauma displayed higher TMD indexes and had a longer duration of symptoms, as well as greater somatization, depression, anxiety, phobic anxiety, and paranoid ideation than did those without a history of trauma^34^. In addition, adolescents with posttraumatic stress disorder demonstrated increased somatization, interpersonal sensitivity, obsessive-compulsiveness, depression, anxiety, and phobic anxiety over time^46^.

Using linear regression analysis, this relationship was further quantified in our study; a higher score for these factors was associated with the presence of a history of trauma in young adolescents. Psychological factors have been widely recognized to be involved in the pain perception process in children and adolescents^47^. Although the etiology of TMD according to age in adolescents is unclear, psychological factors have been implicated in the predisposition, initiation, and perpetuation of TMD^48,49^. In general, individuals with TMD pain exhibit greater psychological maladjustment than do healthy controls^46^. Moderate to severe somatization was observed in approximately 60% of patients with TMD^50^. The individuals who were unable to cope well with TMD demonstrate higher rates of somatization and depression^51^. Unfortunately, our study did not include a comparison of healthy adolescents and adolescents with TMD. Nevertheless, we explored the psychological factors playing a role in adolescents with TMD and a history of trauma, which has rarely been done previously. Thus, a history of trauma can exacerbate psychological factors associated with TMD in young adolescents.

To understand the psychological factors related to pain and trauma in young adolescents, we must consider the multidimensional nature of adolescent TMD, within the context of the biopsychosocial model, because adolescents are vulnerable in terms of functional TMJ pain derived from the interplay between organic dysfunction and psychosocial factors^52,53^. Psychological stress resulting from events occurring at school and in the family, and the related muscle hyperactivity and muscle fatigue, as well as oral habits, have been suggested as etiological factors^2^. Clinically, hypervigilance and hypersensitivity often cause heightened awareness of pain in young adolescents^54^. Therefore, young adolescents describe more pain on muscle palpation and have a heightened fear of being touched. Further research is needed to understand the complex biobehavioral processes involved in adolescent TMD.

In addition to our strengths, our research has several limitations. First, this study is fragmentary with cross-sectional observational study design. With this design, it was not possible to clarify the change over time of the variable or the causality between the variables, only to examine the fragmentation state or correlations. Second, although randomly selected patients were included, the sample size was small (n = 70). Due to the nature of clinical studies with MRI-based diagnosis, there was a practical limitation that the number of samples could not be as large as LeResche *et al*.’s cohort studies on predictors for TMD pain in adolescents with outstanding and important results^55,56^. Conversely, we performed direct observation of the patient rather than conducting telephonic interviews, and the patient was diagnosed by experienced dentists and radiologists, and not hygienists. Furthermore, we conducted statistical power analyses using G*Power 3.1^57^, and obtained reliable power values of the main variables, at above 80%. Finally, we investigated many variables for the limited number of patients. Some variables were related, and the hypotheses for each test were not completely independent in our study, possibly increasing statistical type I errors. We also simply compared the values of two TMD groups, not more than three. Thus, the Bonferroni corrections, known as a strict and conservative method of reducing type I errors but increasing type II errors^58^, was not implemented. Nevertheless, further longitudinal cohort studies with large samples are needed to confirm and extend our findings.

## Conclusions

No previous studies have simultaneously investigated the characteristics of clinical, psychological, and MRI findings in adolescents with TMD and their comparison according to the presence and absence of trauma. The multidimensional nature of adolescent TMD can be best considered within the context of the biopsychosocial model. Our study emphasized that a history of trauma may be a key factor for increased psychological dimensions in this condition. Furthermore, our findings provide evidence that measuring psychological functioning in the presence of a history of trauma in young adolescents with TMD may be beneficial in its treatment.

## Supplementary information

Consort checklist

## References

[1] Okeson JP, de Leeuw R (2011). Differential diagnosis of temporomandibular disorders and other orofacial pain disorders. Dental clinics of North America.

[2] Sharma S, Gupta DS, Pal US, Jurel SK (2011). Etiological factors of temporomandibular joint disorders. National Journal of Maxillofacial Surgery.

[3] Burakoff RP, Kaplan AS (1993). Temporomandibular disorders: current concepts of epidemiology, classification, and treatment. Journal of pain and symptom management.

[4] LeResche L (1997). Epidemiology of temporomandibular disorders: implications for the investigation of etiologic factors. Critical reviews in oral biology and medicine: an official publication of the American Association of Oral Biologists.

[5] Dworkin SF (1990). Epidemiology of signs and symptoms in temporomandibular disorders: clinical signs in cases and controls. Journal of the American Dental Association (1939).

[6] Dworkin SF, LeResche L (1992). Research diagnostic criteria for temporomandibular disorders: review, criteria, examinations and specifications, critique. Journal of craniomandibular disorders: facial & oral pain.

[7] Steed PA, Wexler GB (2001). Temporomandibular disorders–traumatic etiology vs. nontraumatic etiology: a clinical and methodological inquiry into symptomatology and treatment outcomes. Cranio: the journal of craniomandibular practice.

[8] Gage JP (1985). Collagen biosynthesis related to temporomandibular joint clicking in childhood. The Journal of prosthetic dentistry.

[9] Jaworska N, MacQueen G (2015). Adolescence as a unique developmental period. Journal of psychiatry & neuroscience: JPN.

[10] Nilsson IM, List T, Drangsholt M (2013). Headache and co-morbid pains associated with TMD pain in adolescents. Journal of dental research.

[11] Schiffman E (2014). Diagnostic Criteria for Temporomandibular Disorders (DC/TMD) for Clinical and Research Applications: recommendations of the International RDC/TMD Consortium Network* and Orofacial Pain Special Interest Groupdagger. Journal of oral & facial pain and headache.

[12] Slade GD (2011). Study methods, recruitment, sociodemographic findings, and demographic representativeness in the OPPERA study. J Pain.

[13] Fillingim RB (2011). Potential psychosocial risk factors for chronic TMD: descriptive data and empirically identified domains from the OPPERA case-control study. J Pain.

[14] Østensjø V, Moen K, Storesund T, Rosén A (2017). Prevalence of Painful Temporomandibular Disorders and Correlation to Lifestyle Factors among Adolescents in Norway. Pain Research & Management.

[15] Casey BJ, Jones RM, Hare TA (2008). The adolescent brain. Annals of the New York Academy of Sciences.

[16] Gorska A (2014). Temporomandibular joint dysfunction and disorders in the development of the mandible in patients with juvenile idiopathic arthritis - preliminary study. Advances in clinical and experimental medicine: official organ Wroclaw Medical University.

[17] Shaw L, Morozova M, Abu-Arafeh I (2018). Chronic post-traumatic headache in children and adolescents: systematic review of prevalence and headache features. Pain management.

[18] Nilsson, I. M. Reliability, validity, incidence and impact of temporormandibular pain disorders in adolescents. *Swedish dental journal. Supplement*, 7–86 (2007). 17506471

[19] Nilsson IM, Drangsholt M, List T (2009). Impact of temporomandibular disorder pain in adolescents: differences by age and gender. Journal of orofacial pain.

[20] Cohen LL, Vowles KE, Eccleston C (2010). The impact of adolescent chronic pain on functioning: disentangling the complex role of anxiety. J Pain.

[21] Bonjardim LR, Gaviao MB, Pereira LJ, Castelo PM (2005). Anxiety and depression in adolescents and their relationship with signs and symptoms of temporomandibular disorders. The International journal of prosthodontics.

[22] Krogstad BS, Jokstad A, Dahl BL, Soboleva U (1998). Somatic complaints, psychologic distress, and treatment outcome in two groups of TMD patients, one previously subjected to whiplash injury. Journal of orofacial pain.

[23] Caffo E, Belaise C (2003). Psychological aspects of traumatic injury in children and adolescents. Child and adolescent psychiatric clinics of North America.

[24] Vogl TJ (2016). The value of MRI in patients with temporomandibular joint dysfunction: Correlation of MRI and clinical findings. European journal of radiology.

[25] Su N, Poon R, Friedman L, Darling M, Grushka M (2015). TMJ Changes in Adolescent TMD Patients Seen on MRI in Clinical Setting. The New York state dental journal.

[26] Whyte AM, McNamara D, Rosenberg I, Whyte AW (2006). Magnetic resonance imaging in the evaluation of temporomandibular joint disc displacement–a review of 144 cases. International journal of oral and maxillofacial surgery.

[27] Look JO, Schiffman EL, Truelove EL, Ahmad M (2010). Reliability and Validity of Axis I of the Research Diagnostic Criteria for Temporomandibular Disorders (RDC/TMD) with Proposed Revisions. Journal of oral rehabilitation.

[28] Fricton JR, Schiffman EL (1986). Reliability of a craniomandibular index. Journal of dental research.

[29] Muller L, van Waes H, Langerweger C, Molinari L, Saurenmann RK (2013). Maximal mouth opening capacity: percentiles for healthy children 4-17 years of age. Pediatric rheumatology online journal.

[30] Al-Dlaigan YH, Asiry MA (2014). Maximum mouth opening in saudi adolescents. J Int Oral Health.

[31] Lee Y-H, Lee KM, Auh QS, Hong J-P (2018). Magnetic Resonance Imaging-Based Prediction of the Relationship between Whiplash Injury and Temporomandibular Disorders. Front Neurol.

[32] Cavalcanti RF, Studart LM, Kosminsky M, de Goes PSA (2010). Validation of the multimedia version of the RDC/TMD axis II questionnaire in Portuguese. Journal of Applied Oral Science.

[33] King S (2011). The epidemiology of chronic pain in children and adolescents revisited: a systematic review. Pain.

[34] Kim HI, Lee JY, Kim YK, Kho HS (2010). Clinical and psychological characteristics of TMD patients with trauma history. Oral diseases.

[35] Meeus W, Dekoviic M (1995). Identity development, parental and peer support in adolescence: results of a national Dutch survey. Adolescence.

[36] Palermo TM, Eccleston C (2009). Parents of children and adolescents with chronic pain. Pain.

[37] Rainbow R, Ren W, Zeng L (2012). Inflammation and Joint Tissue Interactions in OA: Implications for Potential Therapeutic Approaches. Arthritis.

[38] Pressman BD, Shellock FG, Schames J, Schames M (1992). MR imaging of temporomandibular joint abnormalities associated with cervical hyperextension/hyperflexion (whiplash) injuries. Journal of magnetic resonance imaging: JMRI.

[39] Garcia R, Arrington JA (1996). The relationship between cervical whiplash and temporomandibular joint injuries: an MRI study. Cranio: the journal of craniomandibular practice.

[40] Bergman H, Andersson F, Isberg A (1998). Incidence of temporomandibular joint changes after whiplash trauma: a prospective study using MR imaging. AJR. American journal of roentgenology.

[41] Sale H, Bryndahl F, Isberg A (2014). A 15-year follow-up of temporomandibular joint symptoms and magnetic resonance imaging findings in whiplash patients: a prospective, controlled study. Oral surgery, oral medicine, oral pathology and oral radiology.

[42] Riggenbach A, Goubert L, Van Petegem S, Amouroux R (2019). Topical Review: Basic Psychological Needs in Adolescents with Chronic Pain-A Self-Determination Perspective. Pain Res Manag.

[43] Gutman LM, Eccles JS (2007). Stage-environment fit during adolescence: trajectories of family relations and adolescent outcomes. Developmental psychology.

[44] Palermo TM, Putnam J, Armstrong G, Daily S (2007). Adolescent autonomy and family functioning are associated with headache-related disability. The Clinical journal of pain.

[45] Chow ET, Otis JD, Simons LE (2016). The Longitudinal Impact of Parent Distress and Behavior on Functional Outcomes Among Youth With Chronic Pain. The journal of pain: official journal of the American Pain Society.

[46] Fillingim, R. B. *et al*. Psychological Factors Associated with Development of TMD: the OPPERA Prospective Cohort Study. *The journal of pain: official journal of the American Pain Society* **14**, 10.1016/j.jpain.2013.1006.1009 (2013). 10.1016/j.jpain.2013.06.009 PMC3855656 24275225

[47] Zernikow B, Hechler T (2008). Pain Therapy in Children and Adolescents. Deutsches Ärzteblatt International.

[48] Sipila K (2001). Association between symptoms of temporomandibular disorders and depression: an epidemiological study of the Northern Finland 1966 Birth Cohort. Cranio: the journal of craniomandibular practice.

[49] Rudy TE, Turk DC, Kubinski JA, Zaki HS (1995). Differential treatment responses of TMD patients as a function of psychological characteristics. Pain.

[50] List T, Dworkin SF (1996). Comparing TMD diagnoses and clinical findings at Swedish and US TMD centers using research diagnostic criteria for temporomandibular disorders. Journal of orofacial pain.

[51] Dworkin SF, Massoth DL (1994). Temporomandibular disorders and chronic pain: disease or illness?. The Journal of prosthetic dentistry.

[52] Adams LM, Turk DC (2015). Psychosocial Factors and Central Sensitivity Syndromes. Current rheumatology reviews.

[53] Vetter TR, McGwin G, Bridgewater CL, Madan-Swain A, Ascherman LI (2013). Validation and Clinical Application of a Biopsychosocial Model of Pain Intensity and Functional Disability in Patients with a Pediatric Chronic Pain Condition Referred to a Subspecialty Clinic. Pain Research and Treatment.

[54] Clinch J, Eccleston C (2009). Chronic musculoskeletal pain in children: assessment and management. Rheumatology (Oxford, England).

[55] LeResche L, Mancl LA, Drangsholt MT, Saunders K, Von Korff M (2005). Relationship of pain and symptoms to pubertal development in adolescents. Pain.

[56] LeResche L, Mancl LA, Drangsholt MT, Huang G, Von Korff M (2007). Predictors of onset of facial pain and temporomandibular disorders in early adolescence. Pain.

[57] Faul F, Erdfelder E, Buchner A, Lang AG (2009). Statistical power analyses using G*Power 3.1: tests for correlation and regression analyses. Behav Res Methods.

[58] Armstrong RA (2014). When to use the Bonferroni correction. Ophthalmic & physiological optics: the journal of the British College of Ophthalmic Opticians (Optometrists).

